# Validity of the Polar V800 Monitor for Assessing Heart Rate Variability in Elderly Adults under Mental Stress and Dual Task Conditions

**DOI:** 10.3390/ijerph18030869

**Published:** 2021-01-20

**Authors:** Chang-Jing Huang, Hsiao-Lung Chan, Ya-Ju Chang, Shu-Mei Chen, Miao-Ju Hsu

**Affiliations:** 1 The Master Program of Long-Term Care in Aging, Kaohsiung Medical University, Kaohsiung 807378, Taiwan; hkhs8523@gmail.com; 2 Kaohsiung LOHAS Home Care Institution, Kaohsiung 82061, Taiwan; 3 Department of Electrical Engineering, College of Engineering, Chang Gung University, Tao-Yuan 33302, Taiwan; chanhl@mail.cgu.edu.tw; 4 Neuroscience Research Center, Chang Gung Memorial Hospital, Linkou 333423, Taiwan; 5 School of Physical Therapy and Graduate Institute of Rehabilitation Science, College of Medicine, Chang Gung University, Tao-Yuan 33302, Taiwan; yjchang@mail.cgu.edu.tw; 6 Department of Physical Therapy, College of Health Science, Kaohsiung Medical University, Kaohsiung 807378, Taiwan; shumei@kmu.edu.tw; 7 Department of Medical Research, Kaohsiung Medical University Hospital, Kaohsiung 807378, Taiwan; 8 Center for Long-Term Care Research, Medical University, Kaohsiung 807378, Taiwan

**Keywords:** validity, heart rate variability, heart rate, autonomic nervous system

## Abstract

*Background*: Aging may result in autonomic nervous dysfunction. Heart rate variability (HRV) is a non-invasive method to measure autonomic nervous activities. Many studies have shown that HRV contributes to the risk assessment of diseases. A Polar V800 heart rate monitor is a wearable device that measures R-R intervals, but has only been validated in younger adults under limited testing conditions. There is no validation of the V800 under mental stress or in dual task testing conditions. Therefore, this study investigated the validity of the Polar V800 heart rate monitor for assessing R-R intervals and evaluated if there were differences on HRV parameters under different situations in community-dwelling elderly adults. *Methods*: Forty community-dwelling elderly adults were recruited. Heart rates were recorded via electrocardiogram (ECG) and the V800 under sitting, during an arithmetic test, during a naming test, a self-selected walking velocity test (SSWV), and dual tasks (SSWV performing mental arithmetic test and SSWV performing naming test). Indices of time and frequency domains of HRV were calculated afterwards. The intra-class correlation coefficient (ICC) analysis and effect size were calculated to examine the concurrent validity between the V800 and the ECG. *Results*: All HRV indices from the V800 were highly correlated with the ECG under all tested conditions (ICC = 0.995–1.000, *p* < 0.001) and the effect size of bias was small (<0.1). *Conclusion*: Overall, the V800 has good validity on the assessment of HRV in community-dwelling elderly adults during sitting, mental arithmetic test, naming test, SSWV, and dual tasks.

## 1. Introduction

With aging, the function of the autonomic nervous system changes significantly [1]. Autonomic dysregulation can increase the risk of chronic diseases, such as cardiovascular disease, and it is associated with cognitive decline in the elderly [2,3,4,5,6]. Thus, it is important to assess the autonomic function in elderly adults.

Heart rate variability (HRV) is a non-invasive measurement of autonomic nerve activity and has been widely used. HRV assessment is commonly performed at rest and under mental or physical stress conditions, such as with the Stroop test or mental arithmetic and naming tests [7,8,9,10]. Recent studies have also tested HRV under dual task situations to assess the response of the autonomic function to stimuli [11,12].

Traditionally, HRV is analyzed using an ECG that captures the heart rate R-R interval to measure autonomic nerve activity. In recent years, wearable devices, such as the Polar V800 and the Polar RS800, have been developed to measure R-R interval, which makes the assessment of autonomic function more convenient and more widely applicable. However, validation studies on these devices to analyze HRV are limited. Giles et al. tested whether the V800 is valid in measuring young individuals at rest, and showed that the V800 has good accuracy in measuring HRV [13]. However, their study only tested younger groups, and the testing conditions were limited to resting supine and standing postures. Daily life involves walking and is associated with dual task situations, such as walking and answering questions at the same time. It is important to test whether the V800 is valid for autonomic measurement while performing dual tasks to enhance its application. The accuracy of R-R interval detection depends on the identification of the cardiac waveform. Physical or psychological stress may increase the risk of arrhythmia, which further influences the detection of R-R intervals [14,15,16,17,18]. Previous studies on the validation of wearable heart rate monitors focused primarily on the physical stress aspect. The validity of the V800 under mental stress or in dual task conditions remains unknown. Therefore, the purpose of this study was to investigate the validity of the V800 for R-R intervals and assess if there were differences in derived HRV parameters under sitting, arithmetic test, naming test, self-selected walking velocity (SSWV), and dual tasks (SSWV performing mental arithmetic test and SSWV performing naming test) in elderly adults. We hypothesized that the V800 would have good validity in various testing conditions.

## 2. Materials and Methods

### 2.1. Participants

Forty community-dwelling elderly adults were recruited and completed this study. Inclusion criteria were (1) 65 years of age or older; (2) able to communicate; (3) able to walk independently on a treadmill; and (4) agree to participate voluntarily. Exclusion criteria were (1) severe neurological diseases, such as Parkinson’s disease, epilepsy, etc.; (2) severe cardiopulmonary disease or arrhythmia; (3) cardiovascular events in the past year; (4) less than 24 points on the Mini-Mental State Examination (MMSE) [19]; (5) taking β-blockers, antiarrhythmic drugs, three cyclic antidepressants and centrally acting antihypertensive drugs and/or receiving hormone replacement therapy; and (6) those who had taken caffeine, alcohol, or strenuous exercise 24 h before the test. This research protocol was reviewed and approved by the Institutional Review Board of the Kaohsiung Medical University Hospital (KMUHIRB-E(I)-20190193) and was performed according to the Declaration of Helsinki. Each participant gave written informed consent before their entry into this study.

### 2.2. Mental Stress Test

In this study, mental arithmetic and naming tests were used to stimulate psychological stress in the participants [20,21]. These neuropsychological tests can effectively induce mental stress expressed as physiological responses, such as an increased heart rate, and can differentiate the severity of cognitive impairment [22,23,24,25,26]. Mental arithmetic is considered an important cognitive activity and involves several complex processes, including identifying quantities, arranging the quantities into formulas, and then conducting psychological comparisons and calculations [27,28]. The arithmetic test showed good validity and test-retest reliability (intra-class correlation = 0.95) [29]. In this study, the mental arithmetic test was delivered in an auditory presentation of random numbers from 0 to 9 with an interval of 2 s between numbers. The participant was asked to continuously name the sum of the last two numbers [21]. The test lasted for 5 min and the percentage of correct answers was calculated afterwards.

The naming test requires effective organization of speech retrieval and recall and cognitive self-monitoring [30]. In addition to sufficient memory, it also requires effective executive functions [31]. The above cognitive skills are closely related to daily life, such as learning new things, remembering the route to and from work, or recalling past events, among others. The naming test demonstrated good validity and reliability (ICC > 0.82) [32]. In this study, five categories (animals, vegetables, fruits, cities, and sports) were included with each category lasting 1 min. The participant was asked to generate orally as many words as possible from each category [20].

### 2.3. HRV

#### 2.3.1. HRV Data Acquisition

Heart rates were measured with the V800 (Polar Electro OY, Kempele, Finland) and the ECG. The V800 was equipped with a watch and a chest-wearing heart rate sensor (Polar H7), which was attached to the chest using a chest strap. The ECG electrodes were placed at the left subclavian fossa (reference), the right subclavian fossa (−), and the V5 position (+). The ECG signal acquisition frequency of the electrocardiogram was set at 1000 Hz. The ECG signal was obtained by an ECG transducer and an analog-to-digital signal converter (Biopac MP100A-CE, BIOPAC Systems, Inc., Santa Barbara, CA, USA), and signal analysis software (AcqKnowledge ver 3.9.1.6, BIOPAC Systems, Santa Barbara, CA, USA) converted the signals into digital data for subsequent analysis. The R-R interval raw data from the ECG and the V800 were manually matched for the start points before further analyses.

#### 2.3.2. HRV Data Management

A median filter with a window size of 3 was applied to find the median of each RR and its nearby neighbors in the R-R interval series. If the absolute difference of the median and the processed RR was greater than 8% of the median, the RR was replaced with the median. Therefore, the spike in the RR series, which was mostly caused by ventricular premature contraction or atrial premature contraction, could be removed to the extent that it was possible. This data processing was implemented and performed using MATLAB 2015b (The MathWorks, Natick, MA, USA) and applied to R-R interval data from the ECG and the V800, separately.

#### 2.3.3. HRV Data Analyses

Time-domain and frequency-domain HRV parameters were calculated using MATLAB 2015b (The MathWorks, Natick, MA, USA). The time-domain parameters included SDNN (ms), RMSSD (ms), NN50 (beats), and PNN50 (%). The frequency-domain parameters included total power (total power, TP ≤ 0.4 Hz), high frequency (HF, 0.15–0.4 Hz), low frequency (LF, 0.04–0.15 Hz), very low frequency (VLF, 0.003–0.04 Hz), normalized low frequency (nLF), normalized high frequency (nHF), and LF/HF ratio.

### 2.4. Procedures

The room temperature was set at 22–23 °C. The participant was asked not to consume drinks containing alcohol or caffeine on the day and the day before the tests and not to eat at least 1–1.5 h before the test. The participant was asked not to exercise prior to the testing day and on the testing day as well.

First, SSWV was determined. The researcher adjusted the treadmill speed until it reached a comfortable speed for the participant [33]. Then, the participant sat quietly for 15 min before HRV testing. The participant received six tests, including sitting, mental arithmetic test, naming test, SSWV, SSWV performing arithmetic test, and SSWV performing naming test. Each test lasted 5 min, and a rest period of 5 min was provided between tests. SSWV and dual tasks were performed on a treadmill. Heart rates were monitored continuously from the V800 and the ECG with the data captured for 5 min of each test condition.

### 2.5. Statistical Analysis

SPSS statistics for Windows (ver 22, IBM, Somers, NY, USA) was used for statistical analyses in this study. The Kolmogorov–Smirnov test was used as the normality test. The intra-class correlation coefficient (ICC) was calculated to examine the correlation between the V800 and the ECG. The Bland–Altman plot was used to show the level of the agreement on R-R intervals between the V800 and the ECG as well as all derived HRV parameters. This graphical method illustrated the mean error score (i.e., V800 monitor–ECG) with ±1.96 standard deviations lines (confidence interval) parallel to the mean difference line. Logarithmic transformation of the data was performed before calculating mean difference and the level of the agreement if the normality distribution was violated. Afterwards, data were presented after antilog transformation. Paired t-tests were used to examine if there were significant differences in HRV parameters between the V800 and the ECG. Statistical significance for all analyses was set at *p* < 0.05. The magnitude of differences was assessed by effect size, which represents the difference of the mean divided by the pooled variance, and was calculated for all derived HRV parameters. Effect sizes of ≤0.2, ≤0.5, and >0.8 were considered as small, moderate, and great differences, respectively [34,35].

## 3. Results

Demographic data of the participants are presented in Table 1. The proportion of women was slightly higher (57.5%). Ninety percent of the participants were less than 70 years of age. The averaged body mass index was 24.17 kg/m^2^ ± 3.70 kg/m^2^, suggesting that the participants were within a normal weight range. The MMSE score was 27.2 ± 3.7 points, suggesting the participant was within a normal cognitive range.

The total combined number of detected R-R intervals and the number of corrected R-R intervals in the testing conditions are presented in Table 2. The percentage of corrected number of R-R intervals was <1% under the testing conditions, which is within the range reported in the literature [36].

**Figure 1:**
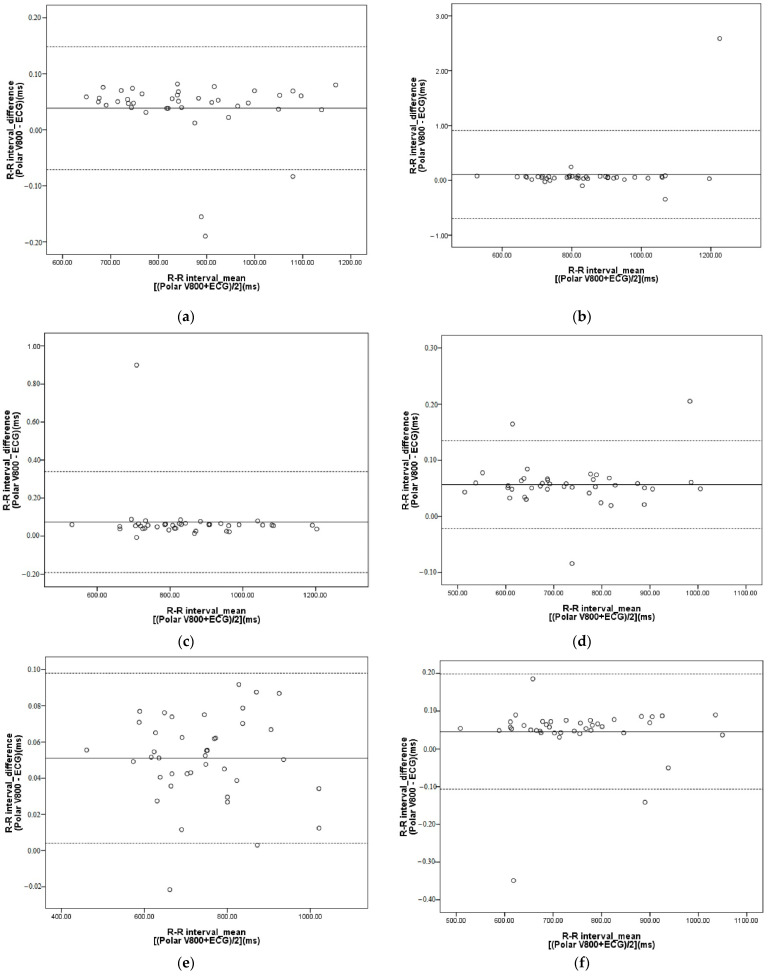
Bland–Altman plot of R-R intervals for the V800 and the ECG. (**a**) Sitting; (**b**) arithmetic test; (**c**) naming test; (**d**) SSWV; (**e**) SSWV performing arithmetic test; (**f**) SSWV performing naming test. Outer dot-dash lines equal ±1.96 standard deviations of the mean.

Table 3, Table 4, Table 5, Table 6, Table 7 and Table 8 show that the ICCs between the V800 and the ECG for time-domain and frequency-domain HRV parameters ranged from 0.995 to 1.000 (*p* < 0.0001) while under the aforementioned testing conditions. As shown in Figure 1, the Bland–Altman plots showed that the bias of R-R intervals for the sitting condition was 0.04ms, and the limit of agreement ranged from −0.07 to 0.15 ms (95% confidence interval). For the arithmetic test, the bias was 0.10 ms and the limit of agreement ranged from −0.70 to 0.91 ms. For the naming test, the bias was 0.07 ms and the limit of agreement ranged from −0.19 to 0.34 ms. For SSWV conditions, the bias was 0.06 ms and the limit of agreement ranged from −0.02 to 0.13 ms. For arithmetic tests at SSWV, the bias was 0.05 ms and the limit of agreement ranged from 0.00 to 0.10 ms. For naming tests at SSWV, the bias was 0.05 ms and the limit of agreement ranged from −0.11 to 0.20 ms. The bias and the limit of agreement for HRV parameters under the testing conditions are presented in Table 3, Table 4, Table 5, Table 6, Table 7 and Table 8. Paired t-tests revealed no significant differences (*p* < 0.05) between the V800 and the ECG for all derived HRV parameters, and the effect size was less than 0.10 under tested conditions (Table 3, Table 4, Table 5, Table 6, Table 7 and Table 8).

## 4. Discussion

Analyzing HRV provides valuable information on autonomic nervous system control, which helps in understanding the health status of elderly adults by predicting the risks for diseases as well as monitoring the effects of training. Traditional R-R interval recording methods used ECG recording, which involves expensive equipment. As technology advanced, wearable devices were developed to provide R-R interval data. However, studies on the validation of these wearable devices remain limited. This study validated the V800 in community-dwelling elderly adults under mental stress tests and dual tasks, and the results suggested that the V800 had good validity.

Previous studies on the validation of wearable devices for evaluating HRV were commonly done under resting conditions or with physical challenges. Nunan et al. tested the validity of the Polar S810 in healthy participants in prone positions and found that the Pearson correlation coefficient ranged from 0.87 to 0.99 for HRV indices (SDNN, RMSSD, LF, HF, and LF/HF ratio) [37]. Similarly, Gamlin et al. found that the Polar S810 was correlated with ECG in sitting and standing positions with the Pearson correlation coefficient ranging from 0.97 to 0.99 [38]. Giles et al. explored the validity of the V800 on HRV under an active orthostatic test, with the results showing high ICCs (>0.99) and a small effect size (<0.1) for all HRV parameters for both supine and standing conditions [13]. The above-mentioned research provided some support for using these wearable devices to assess HRV. Cognitive function declines with age, and this can compromise the social and daily life of elderly adults. Autonomic function is associated with cognitive impairment, and HRV has been considered as a possible early marker for cognitive impairment [4,39]. In this study, we validated the V800 in elderly adults under mental stress using mental arithmetic and naming tests. The results revealed a strong absolute agreement between the V800 and ECG for all time domains and frequency domains for HRV indices, with ICCs ranging from 0.999 to 1.000 and a small effect size ranging from 0.000 to 0.090 under mental stress conditions (arithmetic test and naming test at sitting), which support the use of the V800 to assess HRV under these conditions.

Today, physical and cognitive tasks are concurrently involved in many daily activities. The ability of elderly adults to carry out dual tasks can be impaired, such as walking and talking at the same time [40]. We tested the validity of the V800 in elderly adults under dual task situations and found that the agreement between the V800 and the ECG was significantly high (ICCs = 0.995–1.000, *p* < 0.0001), suggesting that its use to evaluate HRV in elderly adults under a dual task is feasible. Though the use of the chest strap for the V800 could possibly induce artifacts under walking or exercise conditions, this was not the case for our study. Caminal et al. validated the V800 in young healthy individuals during mountain running and showed that the correlations of HRV indices between the V800 and ECG ranged from 0.87 to 1.00 [41]. Hernado et al. tested the RS800 at different levels of exercise intensity based on oxygen consumption and suggested that it had high correlations with ECG for low-frequency components, even for 100% maximal oxygen consumption, but not high-frequency components [42]. However, all HRV indices in this study had high ICCs (0.995–1.000, *p* < 0.0001). No significant differences in any HRV parameters between the V800 and ECG were found, and the effect size was small, ranging from 0.000 to 0.016 under walking conditions (SSWV, SSWV performing arithmetic test, SSWV performing naming test). Slow walking velocity in elderly adults (<2 mph) might explain the inter-study differences.

Kingsley et al. explored the validity of the Polar S810 in the at-rest condition and reported that the bias of agreement on R-R intervals between the Polar 810S and the ECG was less than 1 ms [43], while Gamelin et al. reported a wider bias of 5.89 ms [38]. Similarly, Caminal et al. validated the V800 under running conditions and found that the bias was less than 1 ms [41]. The bias in R-R intervals in this study for all the testing conditions was less than or equal to 1ms, which is within the range reported in the literature. This difference might not be clinically significant in that the effect size in all derived HRV parameters between the V800 and the ECG was small (0.000 to 0.090).

Several limitations should be noted for this study. First, the participants in this study were apparently healthy community-dwelling elderly adults. Future studies should investigate the validity of the V800 in other patient populations. Second, this study only investigated commonly seen time- and frequency-domain HRV indices. Further research is suggested to include other HRV indices, such as entropy, to enhance the application of the V800. Third, due to the study protocol, the respiratory rate could not be controlled in this study. However, since the heart rate was measured by the V800 and the ECG at the same time, the influence of the respiratory rate on the difference of HRV parameters between the two methods might be minimized.

## 5. Conclusions

This study proved that the Polar V800 wearable heart rate monitor has good validity in detecting R-R intervals in community-dwelling elderly adults under mental stress or in dual task conditions. Derived HRV parameters from the V800 are highly consistent with those from ECG.

## Figures and Tables

**Table 1:** Participant characteristics.

Characteristics	Value
Gender (Number (percentage))	
Man	17 (42.5)
Woman	23 (57.5)
Age (y) (Number (percentage))	
65~69	19 (42.5)
70~74	17 (42.5)
75~79	4 (10)
Education level (Number (percentage))	
None	7 (17.5)
Elementary school	16 (40)
Junior high school	3 (7.5)
High school	8 (20)
University	4 (10)
Master’s degree or above	2 (5)
BMI (kg/m^2^) (Mean (SD))	24.17 (3.70)
Systolic blood pressure (mmHg) (Mean (SD))	133.8 (17.2)
Diastolic blood pressure (mmHg) (Mean (SD))	81.0 (14.8)
Heart rate (times/min) (Mean (SD))	71.8 (12.8)
MMSE (points) (Mean (SD))	27.22 (3.66)

**Table 2:** The total number of R-R intervals detected and corrected.

	V800		ECG	
	Collected Points	Corrected Points	Collected Points	Corrected Points
Sitting	14,133	71 (0.5%)	14,133	69 (0.5%)
Mental arithmetic test	14,515	107 (0.7%)	14,515	104 (0.7%)
Naming test	14,520	124 (0.8%)	14,520	124 (0.8%)
SSWV	16,778	52 (0.3%)	16,778	52 (0.3%)
Mental arithmetic test at SSWV	16,536	95 (0.5%)	16,536	94 (0.5%)
Naming test at SSWV	16,348	78 (0.5%)	16,348	76 (0.5%)

Abbreviations: SSWV, self-selected walking velocity; ECG, electrocardiogram.

**Table 3:** HRV parameters for sitting.

		Polar V800 Median (25%; 75%)	ECG Median (25%; 75%)	ICC	*p* Value	Bias (LoA)	Effect Size
Time domain	SDNN (ms)	22.8 (16.8; 26.5)	22.7 (16.8; 26.5)	1.000	<0.0001	0.01 (−0.19 to 0.20)	0.001
	RMSSD (ms)	14.4 (9.2; 24.1)	14.4 (9.1; 23.5)	1.000	<0.0001	0.01 (−0.19 to 0.20)	0.002
	NN50 (beats)	0.0 (0.0; 2.0)	0.0 (0.0; 2.0)	0.999	<0.0001	0.00 (−0.89 to 0.89)	0.000
	PNN50 (%)	0.0 (0.0; 0.7)	0.0 (0.0; 0.7)	0.999	<0.0001	0.00 (−0.34 to 0.34)	0.004
Frequency domain	VLF (ms^2^)	282.7 (166.1; 396.6)	282.6 (166; 396.1)	1.000	<0.0001	1.00 (0.99 to 1.02) *	0.000
	LF (ms^2^)	69.3 (37.8; 126.5)	69.2 (37.5; 126.3)	1.000	<0.0001	1.00 (0.95 to 1.06) *	0.001
	LF (nu)	46.2 (27.5; 67.8)	46.6 (27.8; 69.9)	1.000	<0.0001	0.00 (−0.02 to 0.02)	0.000
	HF (ms^2^)	81.7 (39.7; 197.4)	81.1 (36.7; 191.5)	1.000	<0.0001	1.00 (0.95 to 1.05) *	0.002
	HF (nu)	54.2 (37.3; 74.1)	54.1 (37.2; 74.1)	1.000	<0.0001	−0.00 (−0.01 to 0.01)	0.003
	TP (ms^2^)	508.0 (280.2; 681.4)	506.3 (279.8; 683.0)	1.000	<0.0001	1.00 (0.99 to 1.02) *	0.001
	LF/HF ratio	0.9 (0.4; 1.9)	0.9 (0.4; 1.8)	1.000	<0.0001	1.00 (0.93 to 1.78) *	0.005

* Value presented on a ratio scale after antilog; LoA: limits of agreement.

**Table 4:** HRV parameters for mental arithmetic test.

		Polar V800 Median (25%; 75%)	ECG Median (25%; 75%)	ICC	*p* Value	Bias (LoA)	Effect Size
Time domain	SDNN (ms)	24.3 (18.4; 32.1)	24.3 (18.4; 32.1)	1.000	<0.0001	−0.09 (−1.29 to 1.15)	0.001
	RMSSD (ms)	14.2 (9.5; 21.2)	14.1 (9.5; 21.1)	1.000	<0.0001	−0.09 (−1.29 to 1.11)	0.006
	NN50 (beats)	0.0 (0.0; 1.8)	0.0 (0.0; 1.8)	0.998	<0.0001	−0.05 (−0.93 to 0.83)	0.001
	PNN50 (%)	0.0 (0.0; 0.5)	0.0 (0.0; 0.5)	0.998	<0.0001	−0.02 (−0.32 to 0.28)	0.001
Frequency domain	VLF (ms^2^)	351.3 (165.5; 596.7)	350.9 (165.2; 597.7)	1.000	<0.0001	1.00 (0.98 to 1.02) *	0.001
	LF (ms^2^)	109.5 (61.1; 175.2)	109.6 (61.8; 175.0)	0.999	<0.0001	1.00 (0.98 to 1.01) *	0.001
	LF (nu)	58.2 (45.2; 71.3)	58.5 (45.0; 66.7)	1.000	<0.0001	0.00 (−0.03 to 0.04)	0.019
	HF (ms^2^)	85.3 (41.0; 140.5)	85.2 (40.7; 139.7)	1.000	<0.0001	1.00 (0.95 to 1.04) *	0.008
	HF (nu)	42.7 (32.9; 58.1)	42.7 (35.5; 57.9)	1.000	<0.0001	−0.00 (−0.03 to 0.02)	0.011
	TP (ms^2^)	548.2 (333.2; 973.3)	546.3 (332.4; 963.7)	1.000	<0.0001	1.00 (0.98 to 1.02) *	0.000
	LF/HF ratio	1.4 (0.8; 2.2)	1.4 (0.8; 1.8)	0.999	<0.0001	1.01 (0.87 to 1.15) *	0.011

* Value presented on a ratio scale after antilog; LoA: limits of agreement.

**Table 5:** HRV parameters for naming test.

		Polar V800 Median (25%; 75%)	ECG Median (25%; 75%)	ICC	*p* Value	Bias (LoA)	Effect Size
Time domain	SDNN (ms)	24.6 (18.4; 32.7)	24.6 (18.4; 32.8)	1.000	<0.0001	−0.01 (−0.21 to 0.20)	0.090
	RMSSD (ms)	15.5 (9.9; 21.9)	15.5 (10.1; 21.9)	1.000	<0.0001	−0.01 (−0.21 to 0.20)	0.001
	NN50 (beats)	0.0 (0.0; 4.3)	0.0 (0.0; 3.8)	0.999	<0.0001	−0.08 (−1.01 to 0.85)	0.010
	PNN50 (%)	0.0 (0.0; 1.1)	0.0 (0.0; 0.9)	0.999	<0.0001	−0.03 (−0.34 to 0.28)	0.008
Frequency domain	VLF (ms^2^)	363.2 (176.8; 678.1)	363.7 (176.0; 674.1)	0.999	<0.0001	1.01 (0.90 to 1.07) *	0.011
	LF (ms^2^)	123.4 (61.1; 278.9)	124.5 (61.2; 278.3)	1.000	<0.0001	1.00 (0.97 to 1.03) *	0.003
	LF (nu)	62.1 (53.3; 70.9)	62.3 (53.6; 70.8)	1.000	<0.0001	0.00 (−0.01 to 0.01)	0.000
	HF (ms^2^)	80.3 (32.1; 164.2)	79.3 (32.2; 160.9)	1.000	<0.0001	1.00 (0.96 to 1.04) *	0.002
	HF (nu)	39.1 (29.7; 48.9)	39.1 (30.3; 49.0)	1.000	<0.0001	0.00 (−0.01 to 0.01)	0.000
	TP (ms^2^)	582.6 (332.7; 1021.8)	582.7 (330.6; 1020.5)	1.000	<0.0001	1.00 (0.93 to 1.07) *	0.009
	LF/HF ratio	1.6 (1.1; 2.4)	1.6 (1.1; 2.4)	0.999	<0.0001	1.00 (0.95 to 1.05) *	0.002

* Value presented on a ratio scale after antilog; LoA: limits of agreement.

**Table 6:** HRV parameters for SSWV.

		Polar V800 Median (25%; 75%)	ECG Median (25%; 75%)	ICC	*p* Value	Bias (LoA)	Effect Size
Time domain	SDNN (ms)	18.5 (14.3; 24.9)	18.5 (14.1; 24.9)	1.000	<0.0001	0.01 (−0.07 to 0.09)	0.001
	RMSSD (ms)	10.5 (7.3; 17.2)	10.4 (7.2; 17.2)	1.000	<0.0001	0.01 (−0.07 to 0.09)	0.003
	NN50 (beats)	0.0 (0.0; 1.0)	0.0 (0.0; 1.0)	1.000	<0.0001	0.00 (0.00 to 0.00)	0.000
	PNN50 (%)	0.0 (0.0; 0.3)	0.0 (0.0; 0.3)	1.000	<0.0001	0.00 (−0.24 to 0.24)	0.000
Frequency domain	VLF (ms^2^)	215.7 (125.4; 394.1)	215.5 (126.9; 394.0)	1.000	<0.0001	1.00 (0.99 to 1.00) *	0.001
	LF (ms^2^)	53.7 (25.5; 92.3)	53.3 (25.5; 92.1)	1.000	<0.0001	1.00 (0.96 to 1.04) *	0.004
	LF (nu)	61.4 (46.0; 75.6)	61.8 (46.3; 75.4)	1.000	<0.0001	0.00 (−0.02 to 0.02)	0.004
	HF (ms^2^)	32.8 (17.6; 76.1)	32.8 (17.9; 75.8)	1.000	<0.0001	1.00 (0.95 to 1.06) *	0.000
	HF (nu)	38.9 (29.0; 53.9)	39.0 (28.8; 54.5)	1.000	<0.0001	0.00 (−0.01 to 0.01)	0.003
	TP (ms^2^)	333.6 (200.5; 602.9)	333.9 (194.8; 602.1)	1.000	<0.0001	1.00 (0.98 to 1.02) *	0.000
	LF/HF ratio	1.6 (0.9; 2.4)	1.6 (0.9; 2.6)	1.000	<0.0001	1.00 (0.94 to 1.05) *	0.002

* Value presented on a ratio scale after antilog; LoA: limits of agreement.

**Table 7:** HRV parameters for mental arithmetic test at SSWV.

		Polar V800 Median (25%; 75%)	ECG Median (25%; 75%)	ICC	*p* Value	Bias (LoA)	Effect Size
Time domain	SDNN (ms)	19.5 (13.7; 26.0)	19.5 (13.7; 26.0)	1.000	<0.0001	0.00 (−0.08 to 0.08)	0.000
	RMSSD (ms)	11.0 (7.3; 16.8)	10.8 (7.4; 16.9)	1.000	<0.0001	0.01(−0.23 to 0.24)	0.001
	NN50 (beats)	0.0 (0.0; 1.0)	0.5 (0.00; 1.00)	0.995	<0.0001	0.08 (−1.04 to 1.20)	0.016
	PNN50 (%)	0.0 (0.0; 0.3)	0.1 (0.0; 0.3)	0.997	<0.0001	0.02 (−0.25 to 0.29)	0.015
Frequency domain	VLF (ms^2^)	270.8 (83.7; 524.2)	271.8 (83.7; 523.7)	1.000	<0.0001	1.00 (0.99 to 1.01) *	0.000
	LF (ms^2^)	57.0 (26.0; 103.7)	57.2 (25.8; 103.7)	1.000	<0.0001	1.00 (0.97 to 1.04) *	0.000
	LF (nu)	49.7 (39.2; 70.6)	49.5 (39.7; 70.8)	1.000	<0.0001	0.00 (−0.01 to 0.01)	0.006
	HF (ms^2^)	48.3 (19.3; 97.4)	49.0 (19.5; 97.5)	1.000	<0.0001	1.00 (0.95 to 1.04) *	0.002
	HF (nu)	49.5 (29.6; 61.1)	49.4 (29.4; 60.6)	1.000	<0.0001	0.00 (−0.01 to 0.01)	0.005
	TP (ms^2^)	371.2 (182.7; 665.4)	371.4 (182.1; 665.9)	1.000	<0.0001	1.00 (0.99 to 1.01) *	0.001
	LF/HF ratio	1.0 (0.6; 2.5)	1.0 (0.7; 2.6)	1.000	<0.0001	1.00 (0.56 to 1.44) *	0.004

* Value presented on a ratio scale after antilog; LoA: limits of agreement.

**Table 8:** HRV parameters for naming test at SSWV.

		Polar V800	ECG	ICC	*p* Value	Bias (LoA)	Effect Size
Time domain	SDNN (ms)	20.3 (14.3; 27.5)	20.3 (14.3; 27.4)	1.000	<0.0001	−0.05 (−0.13 to 0.11)	0.001
	RMSSD (ms)	11.5 (7.4; 18.7)	11.6 (7.2; 18.8)	1.000	<0.0001	−0.02 (−0.42 to 0.39)	0.003
	NN50 (beats)	0.0 (0.0; 1.0)	0.0 (0.0; 1.0)	0.999	<0.0001	−0.05 (−1.04 to 0.94)	0.006
	PNN50 (%)	0.0 (0.0; 0.3)	0.0 (0.0; 0.3)	0.999	<0.0001	−0.02 (−0.32 to 0.28)	0.009
Frequency domain	VLF (ms^2^)	226.3 (142.7; 450.0)	226.8 (142.2; 450.2)	1.000	<0.0001	1.00 (0.99 to 1.02) *	0.001
	LF (ms^2^)	68.3 (40.0; 137.0)	68.2 (40.5; 136.5)	1.000	<0.0001	0.99 (0.54 to 1.45) *	0.005
	LF (nu)	58.3 (44.8; 66.1)	58.6 (45.0; 66.1)	1.000	<0.0001	0.00 (−0.01 to 0.01)	0.006
	HF (ms^2^)	45.7 (25.6; 102.3)	45.5 (25.7; 104.0)	1.000	<0.0001	1.00 (0.95 to 1.05) *	0.005
	HF (nu)	41.8 (33.3; 54.2)	41.5 (33.3; 54.1)	1.000	<0.0001	0.00 (−0.01 to 0.02)	0.011
	TP (ms^2^)	401.4 (200.8; 730.7)	401.2 (201.3; 730.0)	1.000	<0.0001	1.00 (0.99 to 1.01) *	0.000
	LF/HF ratio	1.3 (0.9; 1.9)	1.3 (0.9; 1.9)	1.000	<0.0001	0.99 (0.94 to 1.04) *	0.011

* Value presented on a ratio scale after antilog; LoA: limits of agreement.

## Data Availability

The data presented in this study are available on request from the corresponding author.
